# Phospholipase D1 is a critical mediator of neutrophil extracellular trap formation and venous thrombosis

**DOI:** 10.3389/fimmu.2025.1666184

**Published:** 2025-10-21

**Authors:** Ryosuke Aihara, Masatomo Takahashi, Kenji Morino, Keisuke Matsubara, Kazufumi Kunimura, Akihiko Nishikimi, Yoshihiro Izumi, Takeshi Bamba, Yoshinori Fukui, Takehito Uruno

**Affiliations:** ^1^ Division of Immunogenetics, Department of Immunobiology and Neuroscience, Medical Institute of Bioregulation, Kyushu University, Fukuoka, Japan; ^2^ Division of Metabolomics, Medical Research Center for High Depth Omics, Medical Institute of Bioregulation, Kyushu University, Fukuoka, Japan; ^3^ Biosafety Administration Division, Research Institute, National Center for Geriatrics and Gerontology, Aichi, Japan

**Keywords:** phospholipase D1, phosphatidic acid, neutrophil extracellular trap, reactive oxygen species, deep vein thrombosis

## Abstract

Neutrophil extracellular traps (NETs) are a host defense mechanism whereby activated neutrophils release decondensed chromatin and antimicrobial proteins into the extracellular space to trap and kill invading pathogens. While effective in clearing pathogens, NETs also pose pathological risks by exposing self-DNA, histones, granular enzymes, and reactive oxygen species (ROS), contributing to pathologies such as autoimmune diseases, inflammatory disorders, and thrombosis. Here, we identify phospholipase D1 (PLD1), a lipid-signaling enzyme that generates phosphatidic acid (PA), as a critical regulator of ROS generation and NET formation in murine neutrophils. Using both PLD1-deficient neutrophils and a selective inhibitor, we demonstrate that PLD1 is essential for NET release. Notably, exogenous PA alone is sufficient to trigger robust ROS production and NET formation. *In vivo*, PLD1-deficient mice fail to generate ROS in an acute lung inflammation model and are protected from venous thrombosis. These findings identify PLD1 and PA as key upstream regulators of NET formation and suggest that pharmacological inhibition of PLD1 could provide a potential avenue for early intervention in NET-related diseases such as venous thrombosis.

## 1 Introduction

Neutrophil extracellular trap (NET) formation is a distinct antimicrobial defense mechanism in which neutrophils expel decondensed chromatin and granular proteins into the extracellular space, forming web-like structures that entrap and kill pathogens (1, 2). Often suicidal, these structures— enriched with histones, myeloperoxidase (MPO) and neutrophil elastase (NE)—immobilize bacteria, fungi, viruses, and parasites, thereby preventing their dissemination while concentrating antimicrobial factors for efficient pathogen clearance (1, 2). Within the vasculature, NETs serve as scaffolds for platelet adhesion and fibrin deposition, promoting thrombus formation—a process termed immunothrombosis, which, under physiological regulation, helps contain invading pathogens and protects host integrity (3, 4). However, this powerful defense mechanism is double-edged: the extracellular exposure of self-DNA, histones, and cytoplasmic and granular proteins poses pathological risks, causing unwanted inflammation and tissue injury. Indeed, dysregulated or excessive NET formation has been implicated in autoimmune diseases, chronic inflammatory disorders, and arterial and venous thrombosis (3–6).

Despite growing interest in NET biology, the signaling pathways orchestrating their formation remain incompletely understood. A pivotal step in NET release is the production of reactive oxygen species (ROS) by the NADPH oxidase complex NOX2 (2, 7, 8). Neutrophils from patients with chronic granulomatous disease, who lack functional NOX2, fail to form NETs, underscoring the critical role of ROS (7). Mechanistically, ROS promote dissociation of NE from MPO-containing granules and facilitate NE translocation into the nucleus, leading to chromatin decondensation and NET formation (9, 10). While NOX2 activation is known to occur downstream of protein kinase C (PKC), small GTPases, and phosphoinositide signaling in response to diverse stimuli (11), the upstream lipid signaling events that coordinate NOX2-dependent ROS production during NET formation remain poorly characterized.

The phospholipase D (PLD) family of lipid-modifying enzymes, PLD1 and PLD2, hydrolyze membrane phosphatidylcholine (PC) to generate phosphatidic acid (PA), a bioactive lipid mediator involved in multiple cellular processes (12–14). In neutrophils, PLD activity and PA have been implicated in ROS generation in previous studies (12, 14, 15), with PA directly interacting with the p47phox subunit of NOX2 to promote membrane assembly and activation of the oxidase complex (16, 17). More recent studies, largely based on pharmacological inhibition, have suggested a predominant role for PLD1 in regulating ROS generation (18, 19). However, genetic evidence supporting PLD1’s contribution to ROS generation is still lacking, and the involvement of PLD1 in NET formation remains unexplored. Defining the role of PLD1 in these processes is critical for understanding how lipid signaling integrates into the molecular pathways that drive NET formation.

Here, we investigated the roles of PLD1, PLD2, and their enzymatic product PA in the regulation of NET formation. Using neutrophils from gene knockout mice, we show that loss of PLD1 leads to markedly reduced ROS production and NET formation in response to diverse stimuli. Furthermore, exogenously applied PA was sufficient to induce robust ROS generation and NET release, implicating PA as a key mediator of upstream regulation. *In vivo*, PLD1 deficiency resulted in loss of ROS production in the lung following lipopolysaccharide (LPS) challenge, and significantly attenuated deep vein thrombosis (DVT), a NET-associated pathology. Together, these findings identify PLD1 and PA as critical mediators of NET formation, offering new insight into the lipid signaling pathways that underlie NET-associated disorders such as venous thrombosis.

## 2 Materials and methods

### 2.1 Animals

Generation of *Pld1^–/–^* and *Pld2^–/–^* mice has been described previously (20, 21). These mice were fertile, developed normally, and appeared healthy. All animals were bred on the genetic background of C57BL/6J strain (from CLEA Japan), confirmed by PCR genotyping according to established protocols (20, 21), and housed under specific pathogen-free conditions in the animal facility at Kyushu University. All animal studies were approved by the Ethics Committee for Animal Experiments of Kyushu University.

### 2.2 Isolation of neutrophils

Bone marrow neutrophils were isolated from the femurs and tibias of mice by separation on a discontinuous Percoll gradient (62%/81%; 1.13 g/ml; Cytiva), as described previously (22, 23). Blood neutrophils were obtained from fresh blood collected by cardiac puncture of mice anesthetized with isoflurane. The collected blood was mixed with an equal volume of 2% dextran by gentle tumbling and allowed to settle for 45 min at room temperature (RT). The leukocyte-rich supernatant was recovered, carefully overlaid onto Histopaque-1077 (Sigma-Aldrich) and centrifuged at 1,200 ×*g* for 30 min at RT. The pelleted cells were washed once with phosphate-buffered saline (PBS; Gibco), and resuspended with Hank’s Balance Salt Solution (HBSS with calcium, magnesium, no phenol red; Gibco) supplemented with 0.5% bovine serum albumin (BSA) to perform hemolysis.

### 2.3 NET formation

Neutrophils (5 × 10^5^ cells/200 μl) were resuspended in phenol red-free RPMI 1640 medium (Gibco), stimulated with PMA (100 nM; Sigma-Aldrich) or lipopolysaccharide (LPS; 1 μg/ml; Sigma-Aldrich; L2654), and seeded onto poly-lysine-coated 14-mm glass bottom dishes (35-mm, D11131H, Matsunami Glass), as described previously (24). In some experiments, neutrophils were pretreated with FIPI (Sigma-Aldrich F5807), PD98059 (Calbiochem), SB203580 (Calbiochem) or vehicle control (0.2% DMSO) for 1 hr at 37°C prior to stimulation. Cells were then incubated in a humidified incubator at 37°C, 5% CO_2_ for the indicated times, followed by staining with SYTO Green and SYTOX Orange (final 1 μM each; Invitrogen) in the RPMI 1640 medium at RT for 30 min in dark. Fluorescent images were acquired with a laser-scanning confocal microscope (LSM 510 Meta, Carl Zeiss) and analyzed with the ImageJ software to quantify the level of extracellular DNA (intensity of SYTOX Orange).

### 2.4 Liposome preparation

To evaluate the direct effect of phospholipids on NET formation, liposomes were prepared as described previously (22) with slight modifications. Briefly, 6 μl of stock PA (Sigma-Aldrich; P9511; egg yolk lecithin), PC (Avanti; 840051P; from egg), or phosphatidylethanolamine (PE) (Sigma-Aldrich; P7943; from egg yolk), each dissolved in chloroform (10 mg/ml) was dried under nitrogen gas in a 1.5 ml microtube. The dried lipid film was resuspended with 20 μl of HBSS, snap-frozen in liquid nitrogen, and thawed in a 37°C water bath. The freeze and thaw cycle was repeated twelve times until the suspension became semi-transparent, followed by three cycles of microtip sonication (10 sec each) to yield liposomes for immediate use.

### 2.5 Luminescent ROS assays

Neutrophils were resuspended in phenol red-free RPMI 1640 medium at 1 × 10^5^ cells/100 μl and mixed with 10 μl of luminol (150 μg/ml; Sigma-Aldrich). The cell suspension was dispensed into 96-well black plates (Thermo Fisher Scientific, #137101) and incubated for 1 hr at 37°C in dark. PMA (final 100 nM) was then added, and cells were incubated for the indicated times. Chemiluminescence resulting from luminol oxidation by ROS was monitored by an IVIS imaging system (Perkin Elmer) with a 1-min exposure time.

### 2.6 Immunoblot analysis

To verify PLD1 deficiency in *Pld1^–/–^* neutrophils (**Supplementary Figure S1**), total cell lysates were prepared as previously described (24): bone marrow neutrophils (8 × 10^6^ cells) in PBS were mixed with an equal volume of Laemmli sample buffer (125 mM Tris-HCl, pH 6.8, 4% SDS, 20% glycerol, 0.01% bromophenol blue) supplemented with 2 mM EGTA, 1 mM dithiothreitol, and complete protease inhibitors (Roche). Samples were boiled at 98°C for 10 min and resolved by a 5-20% gradient precast SDS-PAGE gel (FujiFilm Wako; 1 × 10^6^ cells per lane), followed by immunoblotting. PLD1 was detected with rabbit anti-PLD1 (Proteintech, #18355-1-AP, 1:1000), and GAPDH was detected as a loading control with rabbit anti-GAPDH (Cell Signaling Technology, #D16H11, 1:1000). Blots were incubated with HRP-conjugated goat anti-rabbit IgG (Multi-rAb HRP-Goat Anti-Rabbit Recombinant Secondary Antibody (H+L), Proteintech, #RGAR001, 1:2000), and developed using Immobilon Crescendo Western HRP substrate (WBLUR0100, Millipore).

### 2.7 Cell viability assays

Cell viability was assessed using the resazurin reduction assay (25). Bone marrow neutrophils (1.5-2.0 × 10^5^ cells/200 μl) resuspended in phenol red-free RPMI 1640 medium were seeded in 96-well black plates (Perkin Elmer, #B&W IsoPlate-96) and incubated with the indicated concentrations of FIPI (0–3000 nM; 0.2% DMSO as vehicle) for 1 hr at 37°C. Resazurin stock solution (10 mg/ml in PBS) was then added at final 100 μg/ml in culture, and cells were further incubated for 2 hr. Fluorescence was measured at 540 nm excitation and 590 nm emission using a microplate reader (EnSight, Perkin Elmer). Cell viability was expressed as the relative increase in fluorescence compared to baseline after a total of 3 hr incubation with FIPI, normalized to the vehicle-treated control (0.2% DMSO).

### 2.8 Flow cytometry

Blood samples were collected from mice by cardiac puncture. Red blood cells were removed using Red Blood Cell Lysis Solution (Miltenyi Biotec). Cells were incubated for 10 min at room temperature with the Fixable Viability Stain reagent (BD Biosciences), washed, and blocked for 10 min on ice with anti-mouse CD16/32 antibody (1:1000; 2.4G2; TONBO Biosciences). Surface staining was performed using FITC-conjugated anti-mouse CD45 (1:100; 30-F11; BD Biosciences), APC-conjugated anti-mouse CD11b (1:100; M1/70; BioLegend), and PE-conjugated anti-mouse Gr-1 (1:100; RB6-8C5; BD Biosciences). Data were acquired using a BD FACSVerse cytometer and analyzed with BD FACSuite software (BD Biosciences).

### 2.9 Lipidomic analysis

Neutrophils (1-2 × 10^6^ cells/100 μl) were incubated in phenol red-free RPMI 1640 medium (Gibco) at 37°C and stimulated with 5 μl of PMA (final 100 nM) for the indicated times. Following incubation, cell suspensions were quickly frozen in liquid nitrogen and stored at −80°C until analyses. Neutrophils were prepared for lipid extraction using methanol extraction with minor modifications (26). Briefly, 100 μl of each sample was mixed with 900 μl of methanol containing internal standards: diacylglycerol (DG) 15:0/18:1 (d_7_), 75 pmol; PC 15:0/18:1 (d_7_), 500 pmol; PE 15:0/18:1 (d_7_), 35 pmol; phosphatidylglycerol (PG) 15:0/18:1 (d_7_), 25 pmol; phosphatidylserine (PS) 15:0/18:1 (d_7_), 100 pmol; PA 15:0/18:1 (d_7_), 50 pmol; phosphatidylinositol (PI) 15:0/18:1 (d_7_), 100 pmol; lysophosphatidylcholine (LysoPC) 18:1 (d_7_), 225 pmol; lysophosphatidylethanolamine (LysoPE) 18:1 (d_7_), 10 pmol; lysophosphatidylglycerol (LysoPG) 17:1, 25 pmol; lysophosphatidylserine (LysoPS) 17:1, 625 pmol; lysophosphatidic acid (LPA) 17:0, 625 pmol; and lysophosphatidylinositol (LysoPI) 13:0, 125 pmol. Samples were vortexed for 1 min, sonicated for 5 min at RT, and incubated on ice for 5 min to precipitate proteins. After centrifugation at 16,000 ×*g* for 5 min at 4 °C, 800 μl of the supernatant was collected into clean tubes. Protein concentration in the pellet was determined using the Pierce BCA Protein Assay Kit (Thermo Fisher Scientific). Supernatants were dried under a stream of nitrogen gas and dissolved in 100 μl of methanol:chloroform (1:1, v/v) for analysis by supercritical fluid chromatography (SFC) coupled with a triple-quadrupole mass spectrometry (QqQMS) (Shimadzu Co.) (27). The conditions for SFC were as follows: column, ACQUITY UPC^2^ Torus DEA column (3.0 mm i.d. × 100 mm, 1.7 μm-particle size, Waters Co.); injection volume, 2 µl; column temperature, 50 °C; mobile phase A, supercritical carbon dioxide; mobile phase B (modifier), make-up pump solvent consisting of methanol and water (95:5, v/v) with 0.1% (w/v) ammonium acetate; flow rate of mobile phase, 1 ml/min; flow rate of make-up pump, 0.1 ml/min; back-pressure regulator, 10 MPa. The gradient conditions were as follows: 1% B, 0–1 min; 1–75% B, 1–24 min; 75% B, 24–26 min; 1% B, 26–30 min. QqQMS parameters were as follows electrospray voltage of 4.0 kV in the positive-ion mode and −3.5 kV in the negative-ion mode; nebulizing gas flow rate, 3 L/min; heating gas flow rate, 10 L/min; drying gas flow rate, 10 L/min; desolvation temperature, 250°C; heat block temperature, 400°C; dwell time, 2 ms; pause time, 2 ms. The optimized MRM (multiple-reaction monitoring) parameters for the lipid molecules are shown in **Supplementary Table S1**. Identification and quantification of the lipid molecules was performed using Multi-ChromatoAnalysT ver.1.3.4.0 (Beforce Co.). Quantitative levels of lipids were calculated using peak areas relative to the respective internal standard (IS) and corrected for the total protein amount of each sample (**Supplementary Table S1**). The metabolomics MS raw data have been deposited in the MB-POST repository with the dataset identifier MPST000083.

### 2.10 *In vivo* ROS assay

Mice were anesthetized, and intratracheally injected with 150 μl of LPS (0.2 mg/ml; 500 μg/kg) or control PBS into the lungs using a 24G × 3/4” indwelling needle (Surflow-flash, Terumo). After 24 hr, 200 μl of luminol solution (50 mg/ml; 500 mg/kg) was administered intraperitoneally. Fifteen minutes later, mice were sacrificed, and whole lungs were excised. Lung chemiluminescence was measured using the IVIS imaging system with a 5-min exposure time.

### 2.11 Immunofluorescence

Following ROS assays, harvested lungs were embedded in O.C.T. Compound (Tissue-Tek; Sakura) and frozen at −80°C. Cryosections (7 μm thick) were mounted on glass slides (MAS-01; Matsunami Glass), dried overnight, washed once with PBS to remove the O.C.T. Compound, and fixed with 4% paraformaldehyde (PFA) for 4 min. Sections were incubated with rat anti-mouse FcγRIII/FcγRII (final 5 μg/ml; BD Biosciences) in 1% BSA/PBS for 2 hr at RT to block Fc receptors, followed by incubation with a primary antibody against MPO (1:500 dilution; Merck Millipore #07-496) for 1.5 hr at RT. After two washes with 0.1% Tween-20/PBS, sections were incubated with Alexa Fluor 488-conjugated goat anti-rabbit IgG (Invitrogen) for 1 hr, then stained with DAPI (1 μg/ml; FUJIFILM Wako) for 5 min. Sections were washed twice with 0.1% Tween-20/PBS, mounted with fluorescence mounting media (Dako), and imaged using a laser-scanning confocal microscope (LSM 510 Meta, Carl Zeiss).

### 2.12 DVT model

A murine model of deep vein thrombosis (DVT) was established as previously described (28, 29). Mice were anesthetized by isoflurane, and the inferior vena cava (IVC) was separated from aorta with the utmost care. To induce stenosis and promote thrombosis, the IVC was aligned with a needle of a 7-0 silk nylon suture as a spacer, and then doubly ligated using a 6-0 silk nylon suture just below the level of the left renal vein. The spacer was subsequently removed to block blood flow. At 36 hr after surgery, thrombi that developed inside the IVC as readily visible, red blood cell–rich masses were excised using micro-dissecting scissors by cutting at two points: immediately below the ligation site of the IVC and at the distal end of the thrombus. Harvested thrombi were transferred into pre-weighed plastic containers and weighed on an analytical balance (METTLER TOLEDO; readability, 0.1 mg). Thrombus length was measured with a ruler graduated in 1-mm intervals.

### 2.13 Statistical analysis

Statistical analyses were performed using GraphPad Prism 9. Normality of data distribution was assessed by the Kolmogorov-Smirnov test. Parametric data were analyzed using unpaired two-tailed Student’s t-test, while nonparametric data were analyzed using Mann-Whitney test. A *p*-value of < 0.05 was considered statistically significant.

## 3 Results

### 3.1 PLD1 is essential for PMA-induced NET formation and ROS production

**Figure 1:**
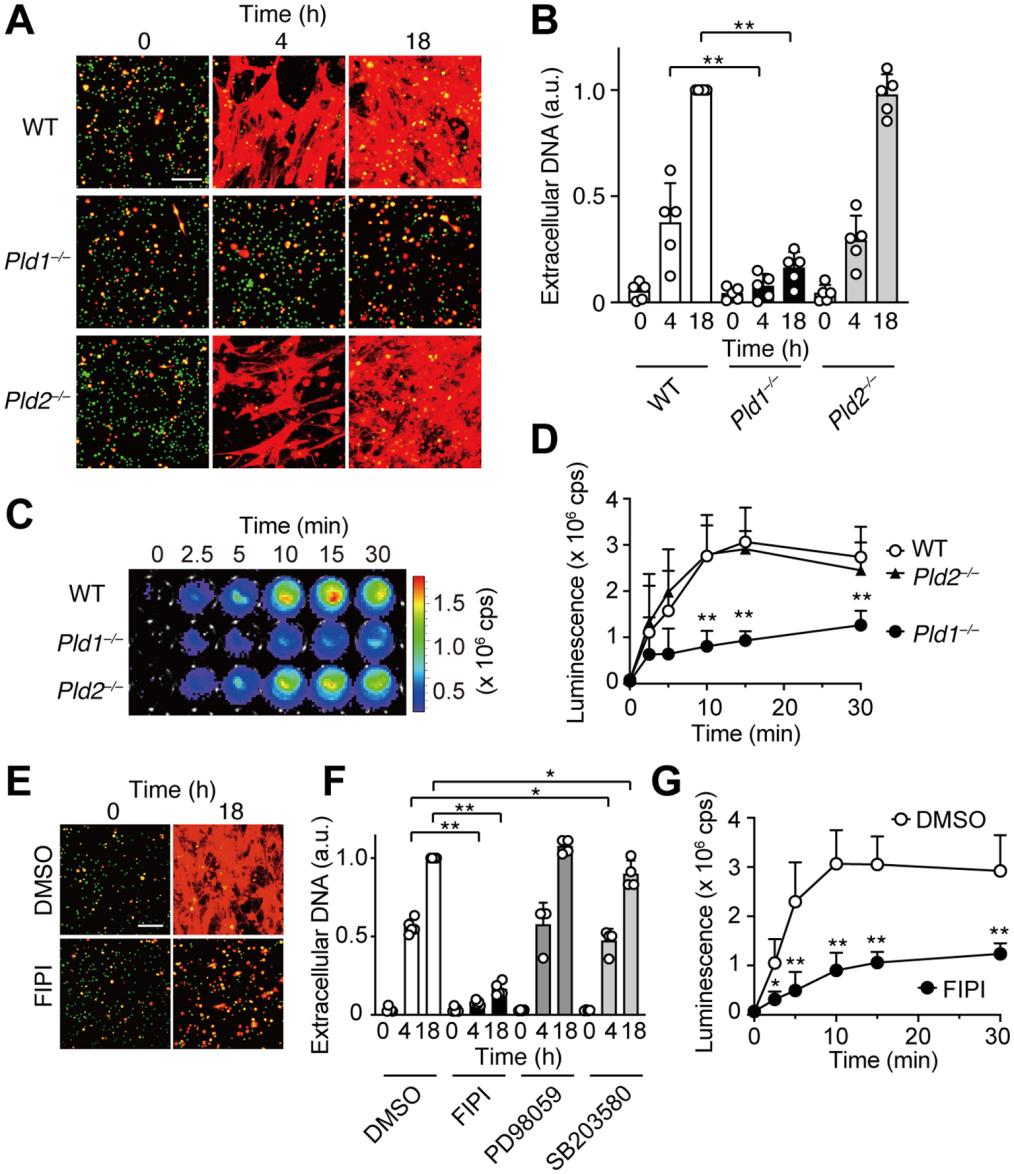
PLD1 is essential for PMA-induced NET formation and ROS production. **(A)** Bone marrow neutrophils from wild-type (WT), *Pld1^–/–^*, and *Pld2^–/–^* mice were treated with PMA (100 nM) for the indicated times. NETs were visualized by co-staining with SYTO Green (cell-permeable, stains intracellular DNA; green) and SYTOX Orange (stains extracellular DNA; red). Representative images are shown. Scale bar, 100 μm. **(B)** Quantification of extracellular DNA. SYTOX Orange fluorescence intensity was measured and normalized to the level of WT neutrophils at 18 hr after PMA treatment. Data are mean ± s.d. (n = 5). **p < 0.01 (unpaired two-tailed Student’s t-test). **(C)** Representative luminescence images showing ROS production. WT, *Pld1^–/–^*, and *Pld2^–/–^* neutrophils were stimulated with PMA (100 nM) in the presence of luminol (150 μg/ml) for the indicated times in a 96-well plate. cps, counts per second. **(D)** Quantification of PMA-induced ROS production in WT, *Pld1^–/–^*, and *Pld2^–/–^* neutrophils. Data are mean ± s.d. (n = 5). **p < 0.01 (unpaired two-tailed Student’s t-test). **(E)** NET formation in WT neutrophils pretreated with FIPI (750 nM) or DMSO (0.1%, vehicle) for 30min prior to PMA stimulation. Representative images are shown. Scale bar, 100 μm. **(F)** Effect of various inhibitors on NET formation. WT neutrophils were pretreated with FIPI (750 nM), PD98059 (50 nM), SB203580 (10 nM), or DMSO (0.1%) for 30min before PMA treatment. Data are mean ± s.d. (n = 5 for DMSO and FIPI; n = 4 for PD98059 and SB203580). *p < 0.05 (Mann-Whitney test), **p < 0.01 (unpaired two-tailed Student’s t-test). **(G)** Effect of FIPI on PMA-induced ROS production in WT neutrophils. Data are mean ± s.d. (n = 5). *p < 0.05, **p < 0.01 (unpaired two-tailed Student’s t-test).

To examine the roles of phospholipase D isoforms PLD1 and PLD2 in neutrophil extracellular trap (NET) formation, bone marrow neutrophils were isolated from wild-type (WT), PLD1-deficient (*Pld1^–/–^*), and PLD2-deficient (*Pld2^–/–^*) mice, and stimulated *in vitro* with phorbol 12-myristate 13-acetate (PMA), a potent NET inducer that activates PKC (7, 8). NETs were visualized by dual staining with SYTO Green, a cell permeable green fluorescent dye that labels both intracellular and extracellular DNA, and SYTOX Orange, a red fluorescent dye that selectively stains extracellular DNA due to its inability to penetrate intact plasma membranes. As shown in **Figure 1A**, PMA stimulation induced robust NET formation in WT and *Pld2^–/–^* neutrophils, evident as prominent red fibrous networks. In contrast, such NET structures were rarely observed in *Pld1^–/–^* neutrophils. Quantification revealed that the extracellular DNA levels in *Pld1^–/–^* neutrophils were markedly reduced, to 20.2% at 4 hr and 16.1% at 18 hr compared to WT neutrophils (**Figure 1B**).

Reactive oxygen species (ROS) play a critical role in PMA-induced NET formation (7, 8, 10). To assess ROS production, neutrophils were stimulated with PMA in the presence of luminol, a chemiluminescent probe that reacts with ROS, enabling its quantitative detection. Upon PMA stimulation, robust ROS generation was observed in WT and *Pld2^–/–^* neutrophils, reaching maximal levels 10–15 min after stimulation. In contrast, ROS production was significantly attenuated in *Pld1^–/–^* neutrophils, averaging approximately 40% of WT levels throughout the time course (**Figures 1C, D**).

To determine whether acute pharmacological inhibition of PLD1 could recapitulate the suppression of NET formation observed in *Pld1^–/–^* neutrophils, we employed FIPI (5-Fluoro-2-indolyl des-chlorohalopemide), a selective cell-permeable inhibitor of both PLD1 and PLD2 (30). FIPI was applied at 750 nM (20, 22, 30), which represents the minimal concentration to achieve maximal inhibition of PLD1 activity in cells (30), and was confirmed to be non-toxic to neutrophils (**Supplementary Figure S2**). Consistent with the results of *Pld1^–/–^* neutrophils, FIPI treatment markedly suppressed PMA-induced NET formation in WT neutrophils, reducing extracellular DNA levels to 13.4% at 4 hr and 16.3% at 18 hr relative to vehicle-treated controls (**Figures 1E, F**). FIPI also significantly attenuated ROS production (**Figure 1G**). In contrast, inhibition of downstream signaling pathways using PD98059 (a MEK inhibitor) or SB203580 (a p38 MAPK inhibitor) had no significant or marginal effect on NET formation (**Figure 1F**). Collectively, these results indicate a crucial role of PLD1 in mediating PMA-induced ROS production and NET formation.

### 3.2 PMA-induced PA production is dependent on PLD1

**Figure 2:**
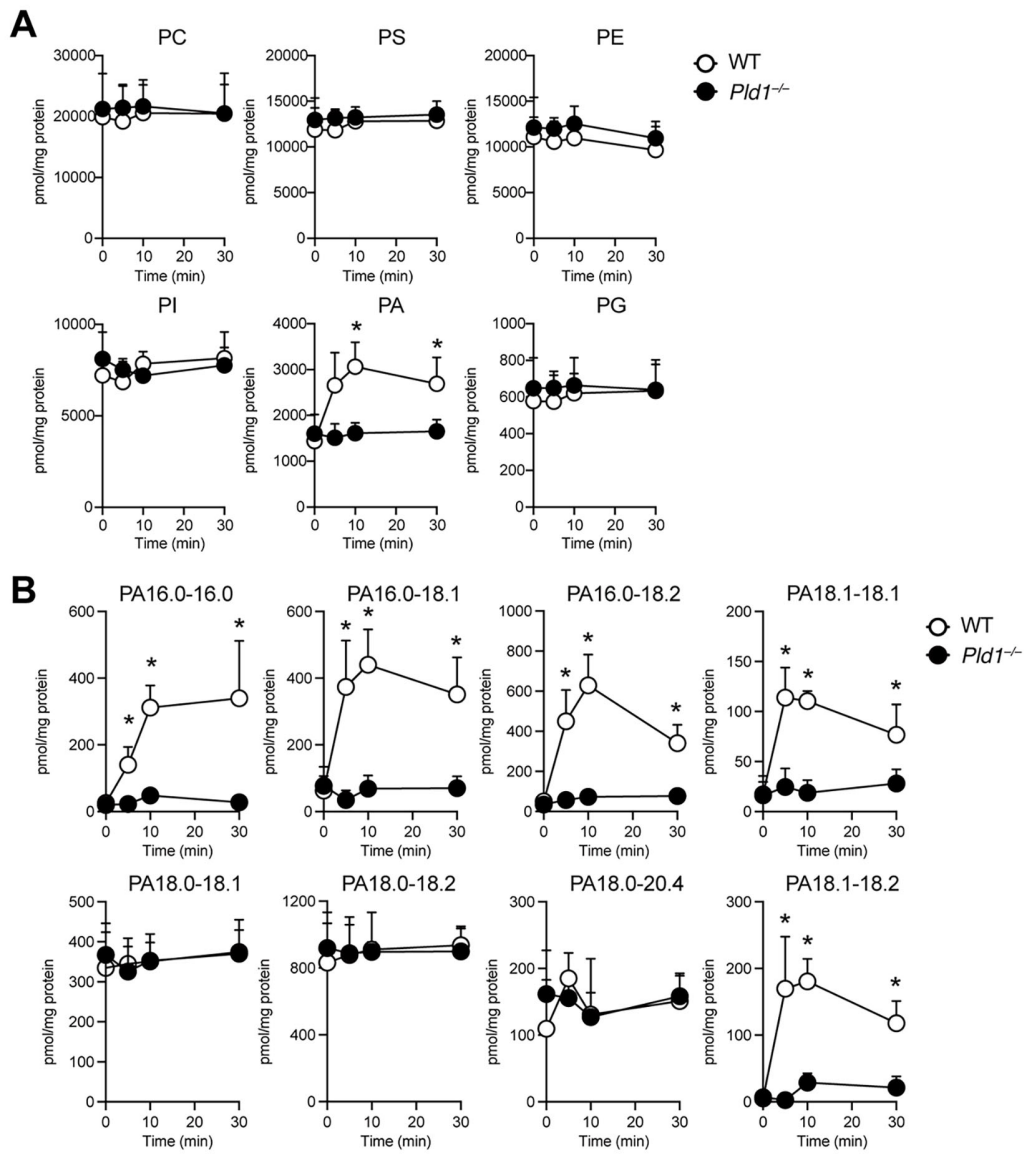
PMA-induced PA production is dependent on PLD1. WT and *Pld1^–/–^* neutrophils were stimulated with PMA (100 nM) for 0, 5, 10, and 30min, and lipid extracts were analyzed for major phospholipid classes **(A)** and individual PA species **(B)**. PC, phosphatidylcholine; PS, phosphatidylserine; PE, phosphatidylethanolamine; PI, phosphatidylinositol; PA, phosphatidic acid; PG, phosphatidylglycerol. Data are presented as mean ± s.d. (n =4). *p < 0.05 (Mann-Whitney test).

Next, we examined changes in phospholipid composition following PMA stimulation in WT and *Pld1^–/–^* neutrophils using mass spectrometry, which we optimized for a highly selective and sensitive identification and quantification of phospholipid species (26, 27). The overall levels of major plasma membrane phospholipids, including PC, PS, PE, PI, and PG, remained largely unchanged in both genotypes (**Figure 2A**). In contrast, a significant increase in PA was observed following PMA stimulation in WT neutrophils. This PMA-induced PA elevation was absent in *Pld1^–/–^* neutrophils, despite comparable basal PA levels between WT and *Pld1^–/–^* neutrophils. As shown in **Supplementary Figure S3**, the levels of lysophospholipids and DG were also comparable between WT and *Pld1^–/–^* neutrophils, except for LysoPA, which transiently increased following PMA stimulation in WT but not in *Pld1^–/–^* neutrophils. While this LysoPA increase is considered to reflect the PLD1-dependent increase in PA upstream, its kinetics do not align with those of ROS production and NET formation.

Further analysis of PA molecular species revealed the presence of two distinct classes of PA in PMA-treated neutrophils (**Figure 2B**). The first class, comprising palmitoyl (16:0_16:0, 16:0_18:1, 16:0_18:2) and oleoyl (18:1_18:1, 18:1_18:2) PAs, was low in resting neutrophils and increased markedly 5–10 min after PMA stimulation in WT neutrophils, but not in *Pld1^–/–^* neutrophils. The second class, stearoyl PAs (18:0_18:1, 18:0_18:2, 18:0_20:4), was abundant in resting neutrophils and remained largely unchanged following PMA stimulation. These results suggest that PLD1 specifically mediates the rapid induction of distinct PA species downstream of PMA signaling, whereas basal PAs are generated through PLD1-independent mechanisms.

### 3.3 PA alone can trigger NET formation and ROS production

**Figure 3:**
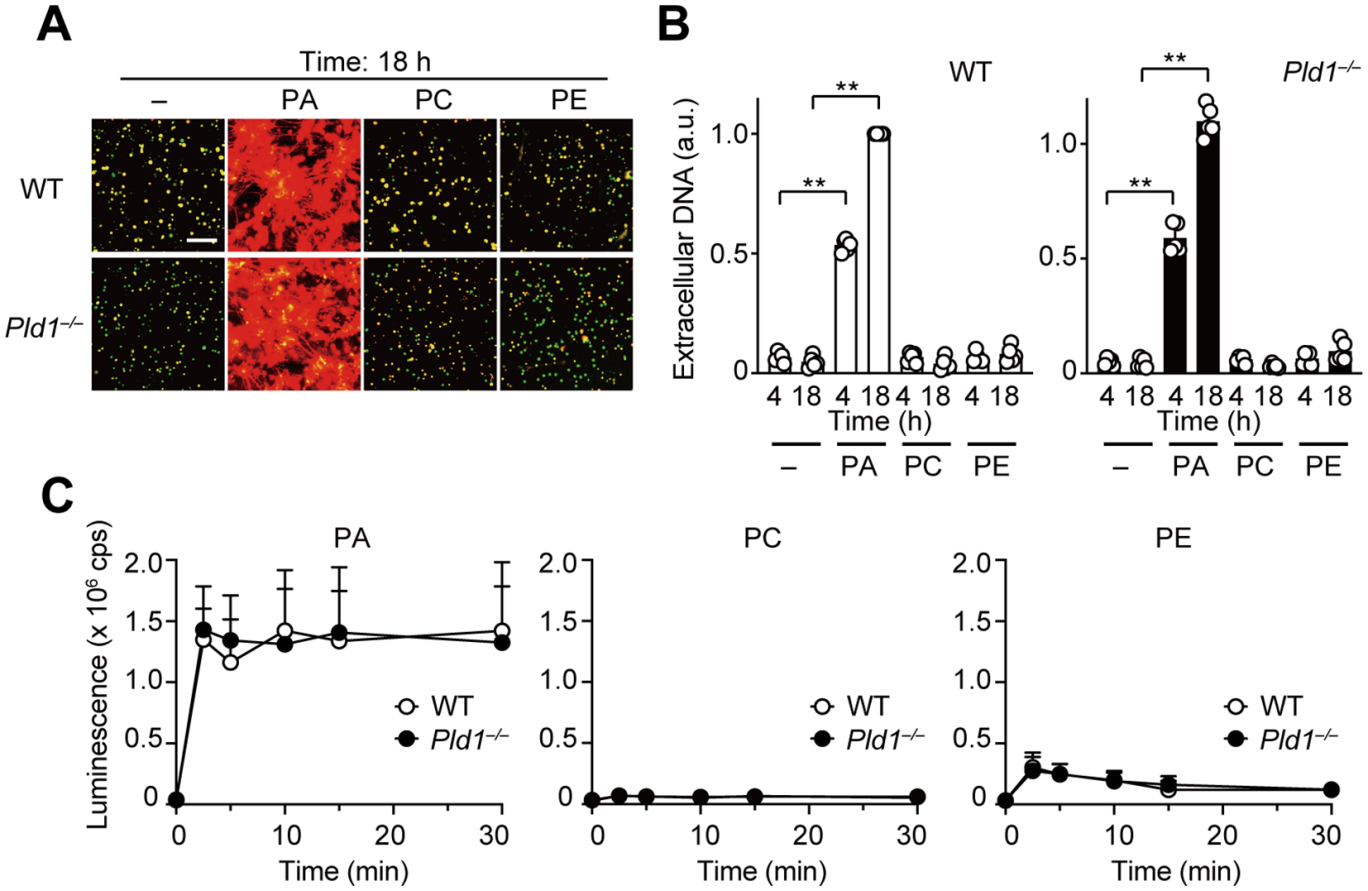
PA is a potent inducer of NET formation and ROS production. **(A)** WT and *Pld1^–/–^* neutrophils were treated with PA, PC, or PE liposomes (final 150 μg/ml) for 18 hr. NETs were visualized by co-staining with SYTO Green (intracellular DNA) and SYTOX Orange (extracellular DNA). Representative images are shown. Scale bar, 100 μm. **(B)** Quantification of extracellular DNA. SYTOX Orange fluorescence intensity was measured and normalized to the level of WT neutrophils treated with PA for 18 hr. Data are mean ± s.d. (n = 5). **p < 0.01 (unpaired two-tailed Student’s t-test). **(C)** Luminescent assay for ROS production in WT and *Pld1^–/–^* neutrophils treated with PA, PC, or PE liposomes. Data are mean ± s.d. (n = 5).

The above results prompted us to investigate whether PA acts as a direct mediator of NET formation. To address this, we directly applied PA-containing liposomes to WT and *Pld1^–/–^* neutrophils. Strikingly, PA liposomes, but not control liposomes composed of PC or PE, induced robust NET formation in both WT and *Pld1^–/–^* neutrophils (**Figures 3A, B**). In line with this, strong ROS production was observed immediately after the addition of PA liposomes in both genotypes (**Figure 3C**), indicating that the cellular machinery required for ROS production and NET formation remains intact in *Pld1^–/–^* neutrophils. Taken together, the results demonstrate that PA alone is sufficient to induce NET formation and ROS production.

### 3.4 PLD1 is essential for LPS-induced NET formation

**Figure 4:**
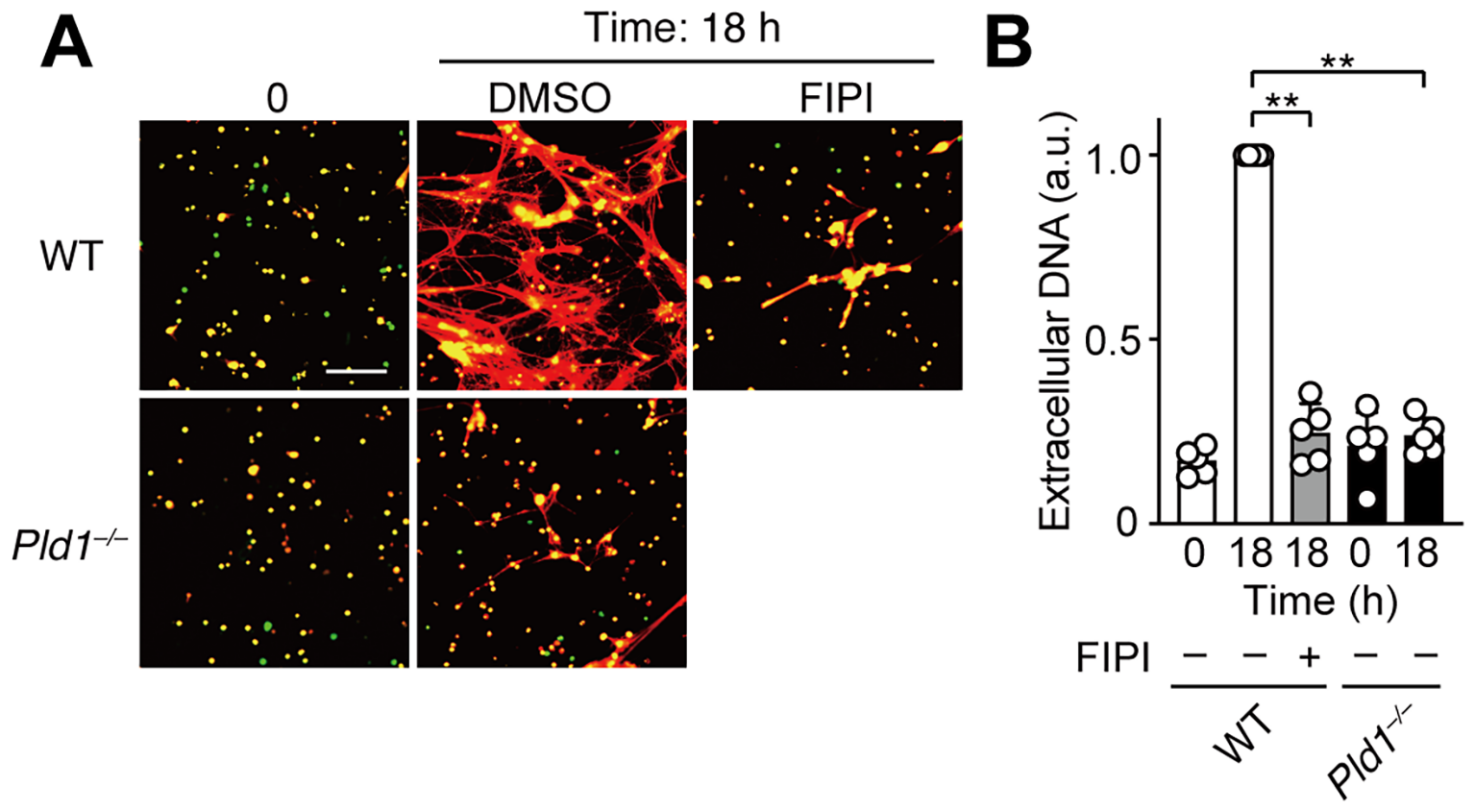
LPS-induced NET formation is suppressed by PLD1 blockade. **(A)** Neutrophils isolated from the peripheral blood of WT and *Pld1^–/–^* mice were pretreated with FIPI (750 nM) or DMSO (0.1%, vehicle) for 30min, followed by stimulation with LPS (1 μg/ml) for 18 hr. NETs were visualized by co-staining with SYTO Green (intracellular DNA, green) and SYTOX Orange (extracellular DNA, red). Representative images are shown. Scale bar, 100 μm. **(B)** Quantification of extracellular DNA. Data are presented as mean ± s.d. (n = 5). **p < 0.01 (unpaired two-tailed Student’s t-test).

LPS, an endotoxin derived from Gram-negative bacteria, has been commonly used as a biologically relevant stimulus for studying NET formation (1, 31). To evaluate the role of PLD1 in this context, we utilized neutrophils isolated from circulating blood, which are more responsive to LPS than bone marrow-derived neutrophils, likely reflecting a differential expression of TLR4 (32, 33). Consistent with previous reports, LPS stimulation induced NET formation in WT neutrophils; however, this response was completely abolished in *Pld1^–/–^* neutrophils (**Figures 4A, B**). Similarly, pharmacological inhibition of PLD1 with FIPI (750 nM) markedly suppressed LPS-induced NET formation in WT neutrophils. Thus, PLD1 activity is critical for LPS-induced NET formation and this response can be effectively suppressed by PLD1 blockade.

### 3.5 PLD1 deficiency suppresses LPS-induced ROS generation *in vivo*

**Figure 5:**
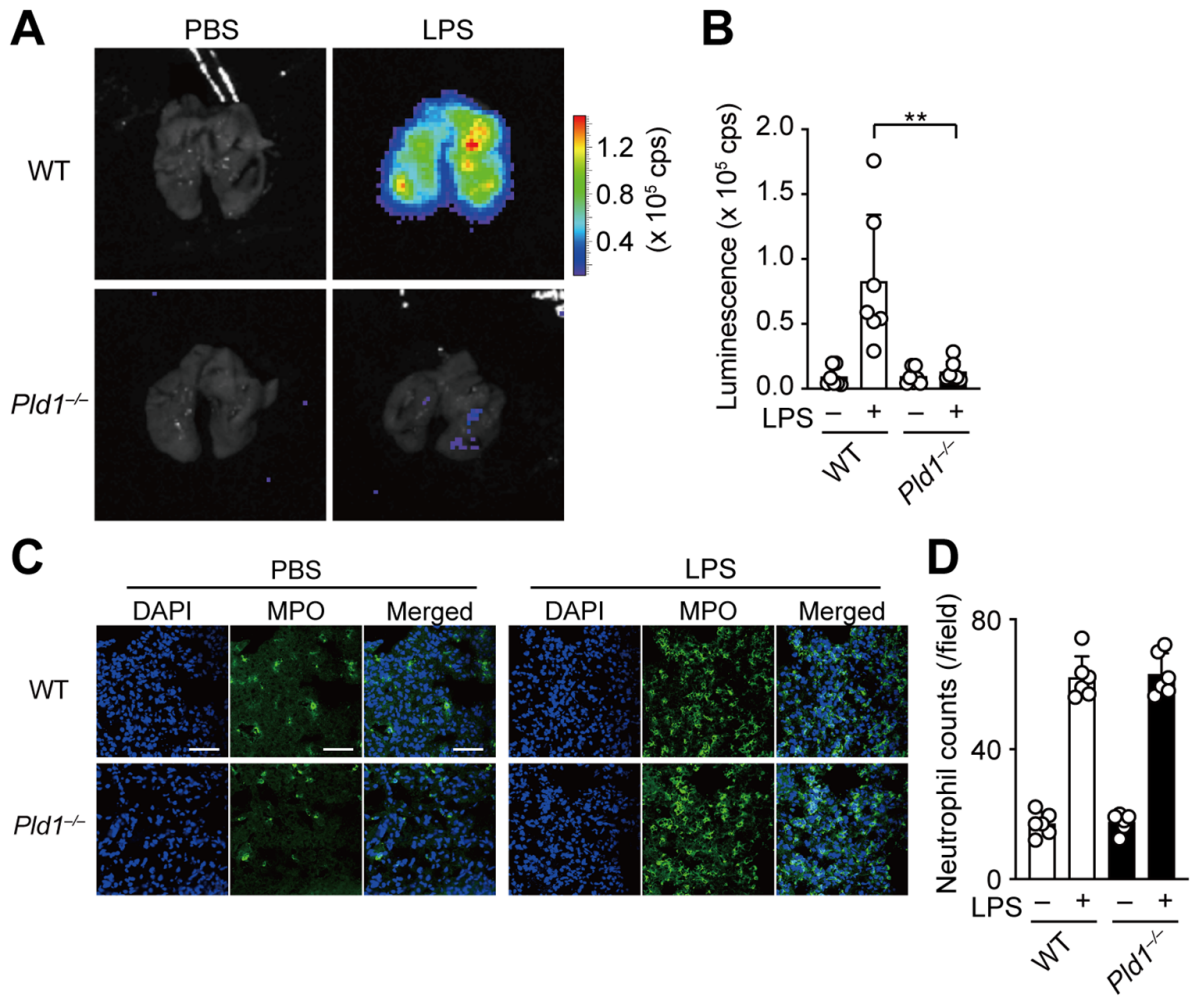
PLD1 is essential for LPS-induced ROS generation *in vivo*. **(A)** WT and *Pld1^–/–^* mice were intratracheally injected with LPS (500 μg/kg) or PBS. After 24 hr, mice received intraperitoneal administration of luminol (500 mg/kg) and were sacrificed 15min later for ROS imaging using the IVIS system. **(B)** Luminescence-based quantification of ROS levels in lungs of WT and *Pld1^–/–^* mice treated as in **(A)**. Data are mean ± s.d. (n = 7 from six independent experiments). **p < 0.01 (Mann-Whitney test). **(C)** Immunofluorescent staining of the lung sections of WT and *Pld1^–/–^* mice treated with PBS or LPS as in **(A)**. Myeloperoxidase (MPO)-positive neutrophils are shown in green; nuclei were stained with DAPI (blue). Scale bar, 50 μm. **(D)** Quantification of neutrophil infiltration in the lung sections of WT and *Pld1^–/–^* mice treated as in **(C)**. Data are mean ± s.d. (n = 7 from six independent experiments).

In a mouse model of acute lung inflammation, intratracheal injection of LPS induces massive infiltration of neutrophils into the lung, where recruited neutrophils serve as the principal source of ROS, contributing to endothelial damage and lung injury (34, 35). To assess the role of PLD1 in neutrophil function *in vivo*, we employed the model and monitored ROS production in the lungs of WT and *Pld1^–/–^* mice 24 hr after LPS administration using IVIS bioluminescence imaging. As expected, LPS injection led to a marked increase in pulmonary ROS levels in WT mice, whereas this response was abolished in *Pld1^–/–^* mice (**Figures 5A, B**). Immunofluorescent staining of lung sections showed that tissue infiltration of neutrophils, defined by myeloperoxidase (MPO) and DAPI double-positive staining, was comparable between WT and *Pld1^–/–^* mice following LPS exposure (**Figures 5C, D**), consistent with previous reports indicating that PLD1 blockade does not blunt or grossly impair neutrophil migration or adhesion (21, 22, 36). Flow cytometric profiling of circulating neutrophils indicated that the frequency of neutrophils in peripheral blood, as well as the surface expression of neutrophil markers (Gr-1, CD11b), was comparable between WT and *Pld1^–/–^* mice (**Supplementary Figure S4**). Quantitatively, the neutrophil fraction in blood showed a 13.3% lower mean value in *Pld1^–/–^* relative to WT mice; however, this difference was not statistically significant (n =5; unpaired t-test, p = 0.8988). These findings indicate that PLD1 deficiency does not overtly disrupt neutrophil abundance in the circulation under steady state conditions. Together, these results demonstrate that PLD1 is dispensable for neutrophil recruitment but is essential for LPS-induced ROS production by neutrophils *in vivo*.

### 3.6 PLD1 deficiency protects mice from developing venous thrombosis

**Figure 6:**
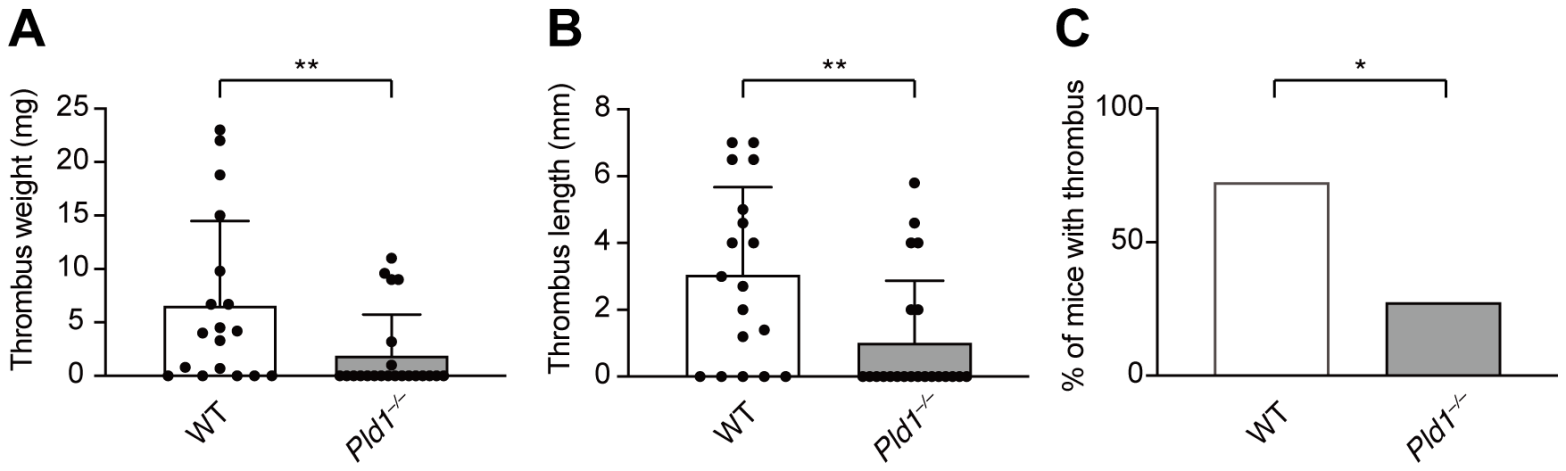
PLD1 deficiency protects mice from developing venous thrombosis. Inferior vena cava (IVC) stenosis was performed on WT and *Pld1^–/–^* mice, and thrombus formation in the IVC was evaluated 36 hr after surgery. Thrombus weight **(A)**, length **(B)**, and incidence of thrombosis **(C)** are shown. Data are presented as mean ± s.d. (WT, n = 18; *Pld1^–/–^*, n = 22 from nine independent experiments). *p < 0.05; **p<0.01 (Mann-Whitney test).

The above results led us to explore the therapeutic potential of PLD1 inhibition in NET-related diseases. Previous studies have established that NETs are key effectors in the pathogenesis of venous thrombosis (4, 37). To examine whether PLD1 deficiency impacts thrombus development, we employed a well-established murine model of deep vein thrombosis (DVT) involving partial flow restriction (stenosis) of the inferior vena cava (IVC) (28, 29). As shown in **Figure 6**, IVC stenosis induced thrombus formation in 72.2% of WT mice within 36 hr post-surgery, whereas only 27.3% of *Pld1^–/–^* mice developed thrombi (*p* = 0.01). Moreover, the extent of thrombosis was significantly attenuated in *Pld1^–/–^* mice, as evident in both thrombus weight (WT: 6.64 ± 7.86 mg; *Pld1^–/–^*: 1.95 ± 3.80 mg; *p* = 0.0068) and length (WT: 3.05 ± 2.62 mm; *Pld1^–/–^*: 1.02 ± 1.85 mm; *p* = 0.048; **Supplementary Figure S5**). Overall, these results indicate that PLD1 deficiency confers protection against DVT, supporting the possibility that inhibiting PLD1 could provide therapeutic benefit in NET-associated thrombotic disorders.

## 4 Discussion

Although NET formation has been extensively studied, the upstream lipid signaling pathways that drive its induction remain incompletely defined. In this study, we identified PLD1 and its lipid product PA as critical mediators of NET formation. Stimulation with PMA markedly increased PA production, accompanied by robust ROS generation and NET release, all of which were abolished in the absence of PLD1. Strikingly, exogenous PA alone was sufficient to elicit ROS production and trigger NET formation. We further demonstrated that LPS-induced NET formation also requires PLD1. *In vivo*, PLD1-deficient mice exhibited impaired ROS production in the lungs following LPS challenge, despite normal neutrophil recruitment, and were protected from deep vein thrombosis (DVT) after IVC stenosis. Together, these findings underscore the crucial role of PLD1 and PA in orchestrating ROS-mediated NET formation, and suggest that inhibiting this pathway could provide therapeutic benefit in NET-associated disorders.

Despite both belonging to the same PLD family, PLD1 is essential for NET formation, whereas PLD2 deficiency has no apparent effect (**Figure 1**). In both human and murine neutrophils, PLD1 is expressed at much higher levels than PLD2 (21, 38). Recent single-cell RNA sequencing data show that in murine bone marrow neutrophils, PLD2 expression is approximately 1/200th of that of PLD1 (Gene Skyline: https://rstats.immgen.org/Skyline/skyline.html; 39). Furthermore, the only other PLD isoform capable of producing PA, PLD6, which catalyzes the conversion of cardiolipin to PA (12, 13), is not expressed in neutrophils (Gene Skyline).

Our lipidomic analysis demonstrated that a distinct class of PA species is generated following PMA stimulation (**Figure 2**). While alternative pathways, such as DGK-mediated phosphorylation of diacylglycerol (DG), can also generate PA, our gene knockout neutrophil data demonstrated that PLD1 is the primary enzyme responsible for PA production under these activated conditions (**Figure 2**). This finding is in line with the fact that PLD1 activity is dynamically regulated by upstream signals, including ARF GTPase, phosphatidylinositol 4,5-bisphosphate, and PKC (12, 13). In contrast, the basal PA pool present in resting neutrophils, which remains largely unchanged upon stimulation, likely originates from the *de novo* phospholipid biosynthetic pathway, where PA serves as an intermediate for generating various classes of phospholipids (40). Notably, these basal PAs differ in their acyl-chain composition from those generated by PLD1. This observation suggests a regulatory mechanism that recognizes the sn-1 fatty acid and supports distinct roles for stimulus-induced versus homeostatic PA pools, underscoring the resolution and robust utility of high-precision lipidomic analysis.

PA can directly bind to the second PX domain of the p47phox subunit, thereby facilitating the assembly of the NOX2 complex at the plasma membrane and promoting ROS generation (16, 17). Consistent with this, a systematic pharmacological study by Ellson et al. (19) showed that FcγR-dependent extracellular ROS production requires PLD1 signaling. In resting cells, PLD1 is mainly localized to the Golgi and intracellular vesicles; however, upon stimulation, it translocates to the plasma membrane, where it generates PA (12, 13, 41). Because PA can modulate membrane curvature and regulate vesicle trafficking through fusion and fission events (14, 41), we speculate that PLD1-derived PA at the plasma membrane may facilitate its microdomain interactions with azurophilic granules over time, thereby coupling NOX2-derived ROS production to the MPO-dependent NE dissociation. Future studies focusing on the intracellular localization of PLD1/PA will be essential to elucidate precisely how PLD1-generated PA can translate to the ROS-dependent downstream events leading to NET formation.

Interestingly, Ellson et al. (19) also demonstrated that phagocytosis-dependent intracellular ROS production (within phagosomes) is PLD1-independent but relies on phosphatidylinositol 3’ kinase (PI3K) signaling. In this setting, PI3K generates phosphatidylinositol 3-phosphate (PI3P), which binds the PX domain of the p40phox subunit and recruits it to the phagosomal membrane for NOX2 assembly. Building on this paradigm, our findings raise the intriguing possibility that selective inhibition of PLD1 may suppress extracellular ROS generation and NET formation without substantially compromising phagocytosis. Indeed, previous studies have suggested that neutrophils can prioritize either phagocytosis or NET formation through distinct regulatory mechanisms (42–44). How ROS generation is differentially coordinated by these two lipid signaling pathways, remains a critical unsolved question that warrants further investigation.

Moreover, we previously identified PA as a potent enhancer of DOCK2, a guanine nucleotide exchange factor critical for Rac activation in neutrophils (22). Notably, DOCK2 is indispensable for both ROS production and NET formation (24). Thus, PA may exert a multifaceted role in promoting NOX2-driven ROS production, by supporting Rac activation via DOCK2 and facilitating NOX2 complex assembly via p47phox. This dual mechanism could represent a unique regulatory axis specific to NET formation.

Aberrant NET formation underlies pathological thrombosis in DVT and has emerged as a contributing factor in inflammatory and thrombotic diseases, including sepsis and COVID-19 (3, 4, 45, 46). Current clinical-stage inhibitors such as DNase I and PAD4 (peptidylarginine deiminase 4) inhibitors act downstream by degrading extracellular DNA or preventing chromatin decondensation (28, 47), but they fail to suppress upstream ROS production, a critical trigger for NET release. Our findings indicate that PLD1 and its product PA function as upstream regulators of NET formation, raising the possibility that pharmacological PLD1 inhibition could modulate NETs at an early stage. Selective PLD1 inhibitors have been developed in the oncology field (20, 48, 49), and PLD1-deficient mice display no major developmental abnormalities (20, 50), although caution is advised given reports of congenital heart defects in patients with biallelic loss-of-function variants in *PLD1* (51). While our data suggest that neutrophil counts are unaffected by PLD1 ablation, the broader impact of systemic inhibition on neutrophil function and host defense requires further study. Moreover, direct comparison of PLD1 and PLD2 in the same thrombosis model will be important to clarify isoform specificity and potential redundancy. In summary, pharmacological PLD1 inhibition could represent a promising strategy for early intervention in NET-associated pathologies.

## Data Availability

The datasets presented in this study can be found in online repositories. The names of the repository/repositories and accession number(s) can be found in the article/**Supplementary Material**.
