# Supplementary material for: Phospholipase D1 is a critical mediator of neutrophil extracellular trap formation and venous thrombosis
Source: Front Immunol. 2025 Oct 21;16:1666184. doi: 10.3389/fimmu.2025.1666184 (PMC12583085; doi:10.3389/fimmu.2025.1666184)
Supplement: Supplementary file 1 [file DataSheet1.pdf]

Supplementary Materials

Supplementary Table:

Table S1. Quantitative lipidomic profiles of PMA-stimulated murine bone marrow neutrophils.

Supplementary Figures:

Figure S1. Immunoblot analysis of PLD1 expression in WT and *Pld1*$^{-/-}$ neutrophils.

Figure S2. Effect of FIPI on neutrophil viability.

Figure S3. Profiles of lysophospholipids and diacylglycerol in WT and *Pld1*$^{-/-}$ neutrophils following PMA stimulation.

Figure S4. Flow cytometric analysis of circulating neutrophils in WT and *Pld1*$^{-/-}$ mice.

Figure S5. PLD1 deficiency attenuates venous thrombus development following IVC stenosis.

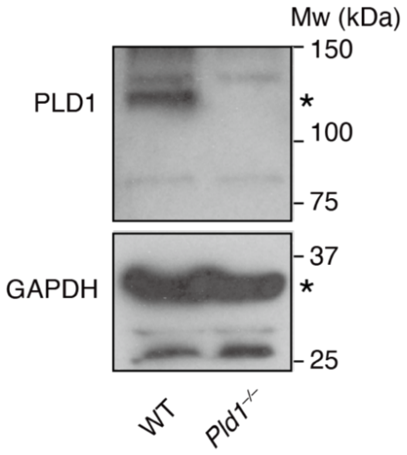

Figure S1. Immunoblot analysis of PLD1 expression in WT and *Pld1*$^{-/-}$ neutrophils. Total cell lysates of bone marrow neutrophils were subjected to immunoblotting with anti-PLD1 and anti-GAPDH (loading control) antibodies. Asterisks (*) indicate the positions of PLD1 and GAPDH proteins, respectively. Molecular weight markers (kDa) are shown on the right.

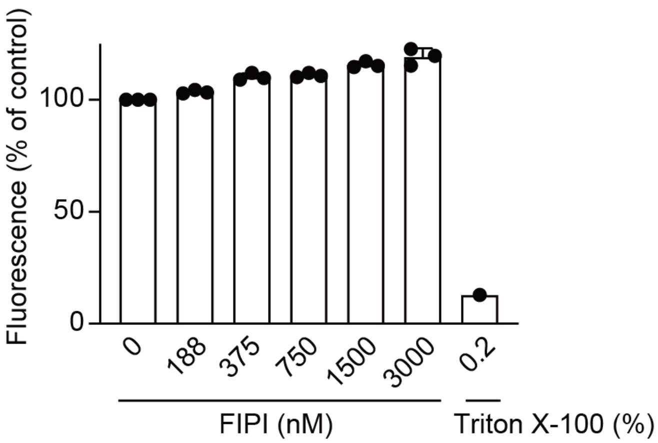

Figure S2. Effect of FIPI on neutrophil viability. WT neutrophils were incubated with the indicated concentrations of FIPI (0–3000 nM; 0.2% DMSO as vehicle), and cell viability was assessed by resazurin reduction assay, measured as the increase in fluorescence after 3 hr. Treatment with 0.2% Triton X-100 served as a positive control for cell lysis (n =1). Data are presented as mean ± s.d. from three independent experiments, each performed in triplicate.

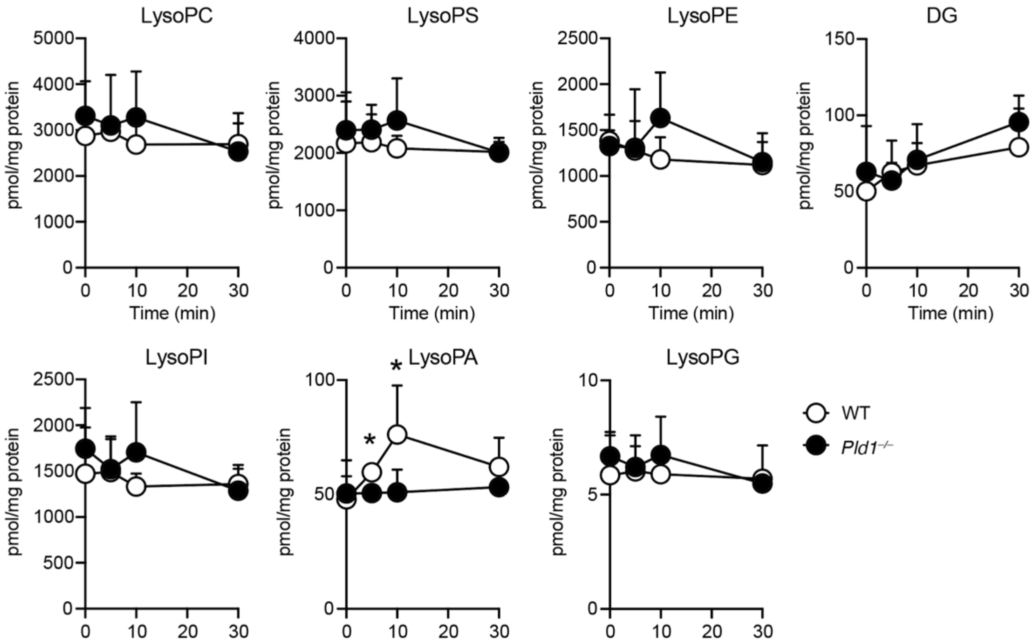

Figure S3. Profiles of lysophospholipids and diacylglycerol in WT and *Pld1*$^{-/-}$ neutrophils following PMA stimulation. Neutrophils were stimulated with PMA (100 nM) for 0, 5, 10, and 30 min, and lipid extracts were analyzed for lysophospholipids and diacylglycerol contents. LysoPC, lysophosphatidylcholine; LysoPS, lysophosphatidylserine; LysoPE, lysophosphatidylethanolamine; DG, diacylglycerol; LysoPI, lysophosphatidylinositol; LysoPA, lysophosphatidic acid; LysoPG, lysophosphatidylglycerol. Data are presented as mean ± s.d. (n =4). *$p < 0.05$ (Mann-Whitney test).

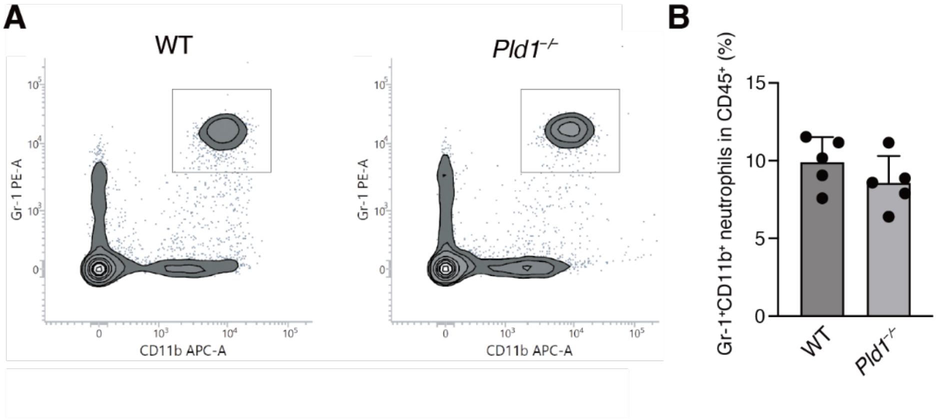

Figure S4. Flow cytometric analysis of circulating neutrophils in WT and *Pld1*$^{-/-}$ mice. (A) Representative profiles from peripheral blood. Neutrophil were defined as CD11b^{+}Gr-1^{+} cells within the CD45^{+} population. (B) Quantification of neutrophil frequency in blood (n = 5; mean ± s.d.; unpaired t-test, p = 0.8988).

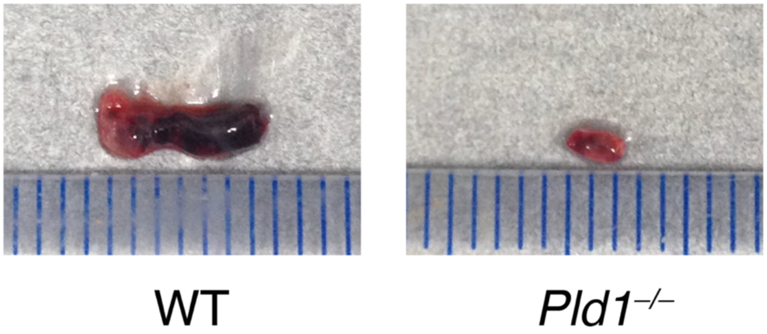

Figure S5. PLD1 deficiency attenuates venous thrombus development following IVC stenosis. Representative images of thrombi formed in WT and *Pld1*$^{-/-}$ mice after partial restriction of the inferior vena cava (IVC). Scale, 1-mm intervals.
